# Density-based hierarchical clustering of pyro-sequences on a large scale—the case of fungal ITS1

**DOI:** 10.1093/bioinformatics/btt149

**Published:** 2013-03-28

**Authors:** Marco Pagni, Hélène Niculita-Hirzel, Loïc Pellissier, Anne Dubuis, Ioannis Xenarios, Antoine Guisan, Ian R. Sanders, Jérôme Goudet, Nicolas Guex

**Affiliations:** ^1^Vital-IT Group, SIB Swiss Institute of Bioinformatics, 1015 Lausanne, Switzerland, ^2^Occupational Environment Department, Institute for Work and Health, Universities of Lausanne and Geneva, 1066 Lausanne, Switzerland and ^3^Department of Ecology and Evolution, University of Lausanne, 1015 Lausanne, Switzerland

## Abstract

**Motivation:** Analysis of millions of pyro-sequences is currently playing a crucial role in the advance of environmental microbiology. Taxonomy-independent, i.e. unsupervised, clustering of these sequences is essential for the definition of Operational Taxonomic Units. For this application, reproducibility and robustness should be the most sought after qualities, but have thus far largely been overlooked.

**Results:** More than 1 million hyper-variable internal transcribed spacer 1 (ITS1) sequences of fungal origin have been analyzed. The ITS1 sequences were first properly extracted from 454 reads using generalized profiles. Then, otupipe, cd-hit-454, ESPRIT-Tree and DBC454, a new algorithm presented here, were used to analyze the sequences. A numerical assay was developed to measure the reproducibility and robustness of these algorithms. DBC454 was the most robust, closely followed by ESPRIT-Tree. DBC454 features density-based hierarchical clustering, which complements the other methods by providing insights into the structure of the data.

**Availability:** An executable is freely available for non-commercial users at ftp://ftp.vital-it.ch/tools/dbc454. It is designed to run under MPI on a cluster of 64-bit Linux machines running Red Hat 4.x, or on a multi-core OSX system.

**Contact:** dbc454@vital-it.ch or nicolas.guex@isb-sib.ch

## 1 INTRODUCTION

Environmental microbiology has advanced greatly in the past few years with the advent of next-generation sequencing technologies. A microbial community can now be sampled by the exhaustive sequencing of the PCR products obtained from a carefully chosen pair of primers. Currently, the Roche 454 pyro-sequencing is the favored technology because it produces relatively long and sufficiently numerous reads at an acceptable cost. However, *pyro-sequences* suffer from a non-negligible rate of error (Huse *et al.*, 2007), which often affects the sequence regions in which a given nucleotide is repeated several times.

In this study, we have used a dataset of the fungal internal transcribed spacer 1 (ITS1) coming from soil samples obtained from 198 different locations in the western Swiss Alps. Each location represented a 4 m^2^ of grassland. The locations were selected by following a random-stratified design and, thus, are distributed over a great variety of soil types and altitudes. Overall, more than a million pyro-sequences have been obtained, with the aim of identifying how biotic and abiotic factors influence the fungal diversity at different scales. Ecological results of this study will be reported elsewhere, the focus of this article being to introduce a novel classification method capable of robustly analyzing this large-scale dataset.

There are currently three taxonomy-independent, i.e. unsupervised, clustering algorithms that can determine Operational Taxonomic Units (OTUs) on a large scale, say in the range of a million of pyro-sequences. These algorithms are cd-hit-454 (Niu *et al.*, 2010), otupipe (Edgar, 2010; Edgar *et al.*, 2011) and ESPRIT-Tree (Cai and Sun, 2011). They have been recently reviewed and numerically benchmarked (Sun *et al.*, 2012) in an article that constitutes an excellent introduction to the field of taxonomy-independent microbial community analysis and that summarizes what is the state of art in the field.

Here, we introduce DBC454, a density-based hierarchical clustering algorithm that departs on several points from the previously mentioned ones. It was designed with the following requirements in mind: it should (i) determine the optimal number of clusters, (ii) properly separate arbitrarily shaped distributions, (iii) not be affected by the order in which the data are presented, (iv) be robust and resistant to noise (e.g. not assign outliers to clusters), (v) use intuitive-free parameters, (vi) be capable of clustering millions of observations in thousands of clusters and (vii) return a solution in a reasonable amount of time.

We also introduce a new numerical benchmark for unsupervised clustering algorithms, which measures their robustness with respect to the errors that are likely to affect the input sequences.

## 2 METHODS

### 2.1 Source material

Fungal ITS1 sequences were obtained from 198 soil samples gathered from 4 m^2^ plots in grasslands along wide soil and altitudinal gradients in the western Swiss Alps (Dubuis *et al.*, 2012). From each plot, five subsamples of 250 mg soil samples were taken, from which DNA was extracted and used for fungi barcoding using the Roche Genome Sequencer FLX Titanium 454 System. The technical details of this experiment will be described in another paper. In total, 1 649 376 reads were generated, of which 1 152 121 were full-length ITS1 sequences (see below).

### 2.2 Extraction of full-length fungal ITS1

**Fig. 1.**
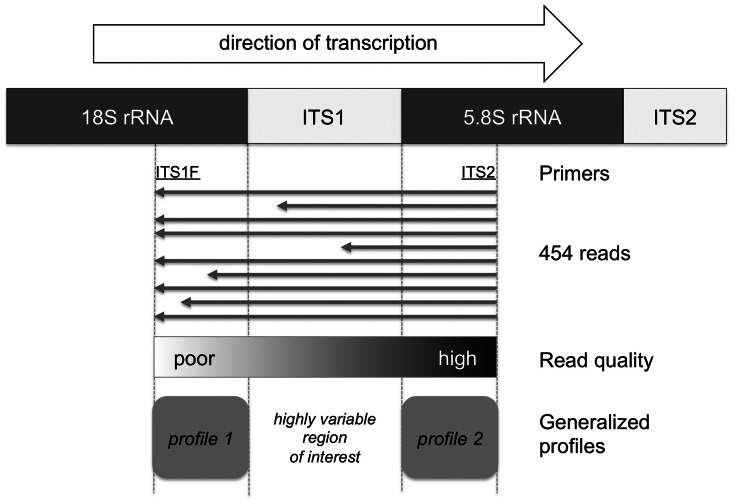
Extraction of ITS1 full-length sequences from the raw 454 reads. Two generalized profiles were built in the two highly conserved regions flanking the ITS1

Two generalized profiles of a length of ∼50 residues were built with the PFTOOLS v2.3 package (Luthy *et al.*, 1994; Sigrist *et al.*, 2002) (http://web.expasy.org/pftools) to recognize the highly conserved regions that flank the ITS1 regions, located at the end of the 18S rRNA and at the beginning of the 5.8S rRNA, respectively (Fig. 1). These profiles were manually edited to produce global matches on the profile side and local matches on the sequence side. In addition, because of the early termination of many pyro-sequences in the 18S region, the profile scoring system at the corresponding extremity (left of profile 1 on Fig. 1) was modified to permit the termination of the alignment at the end of the sequence while within the profile. As far as we know, the generalized profile as implemented in the PFTOOLS v2.3 package remains the only sequence alignment method that permits such a degree of detail in the design of an alignment scoring system for DNA sequences. The two profiles were calibrated on shuffled 454 reads to determine the most appropriate score thresholds (Pagni and Jongeneel, 2001). The pyro-sequences were searched independently with both profiles, and when both were matching on a sequence, the ITS1 was defined as the intervening sequence between them. With this strategy, 1 152 121 full-length ITS1 were recovered, among which 341 585 were unique, out of the 1 649 376 raw pyro-sequences.

The same profile-based search strategy was applied on the FUN, PLN, ENV and INV sections of the release 109 of the EMBL: both strands were searched and 127 577 intervening sequences with a length from 1 to 500 nt were retained as ITS1 to constitute a reference dataset. The biological conclusions drawn with the latter sequences will be discussed in the companion paper.

The only drawback of this profile-based extraction is that it rejects the ITS1 that do not have enough of the 18S region sequenced, i.e. <20 nt. Hence, the longer ITS1 are likely to be under-sampled, given the observed distribution of the lengths of the raw 454 reads (not shown). In contrast, this eliminates the difficulties linked to the analysis of incomplete sequences, which is especially relevant here, as one will only use algorithms that rely explicitly or implicitly on *global sequence alignments*.

### 2.3 Computer programs and parameters

The DBC454 algorithm is presented in Section 3. DBC454 was run using default parameter values (23 steps, using starting and ending distances of 3.317 and 7.141, respectively). We also tested five alternate starting distance levels (3.464, 3.606, 3.873, 4.000 and 4.243). The resulting partitions of the data are named dbc-s0 for the default parameters and dbc-s1 to dbc-s6 for the alternate starting levels.

cd-hit-454 v4.5.4 was run using default parameters, but different values were tested for the sequence identity threshold: 98, 97, 95 and 90%. The resulting partitions of the input data are designated as cdh-98, cdh-97, cdh-95 and cdh-90, respectively.

otupipe v9, with usearch v5.1.221, was run using default parameters, and with different values (98, 97, 95 and 90%) for PCTID_ERR, PCTID_OTU and PCTID_BIN parameters. The resulting partitions of the input data are designated as otu-98, otu-97, otu-95 and otu-90, respectively.

ESPRIT-Tree 11152011 was obtained from the author Yjun Sun and run using default parameters, which produces a hierarchical clustering with 15 levels. By cutting this hierarchy at different heights, one can obtain 15 different partitions of the data. These are named etr-00 to etr-14 (from lower to higher levels).

The sequences of every cluster were aligned using mafft (Katoh and Standley, 2013), and the cluster ‘radius’ was computed as the average of the percent divergences of every sequence pairs in the cluster.

Perl and R scripts were extensively and creatively used throughout this study to re-format, glue together and analyze all the data.

### 2.4 Robustness benchmark

Artificially mutated versions of the ITS1 dataset were created using the msbar program from the EMBOSS suite (Rice *et al.*, 2000). To simulate the errors that occur during the 454 sequencing, we followed the quantitative analysis of Huse *et al.* (2007). Briefly, 18% of the original sequences were randomly selected, and among them, 61% were given a single error, 17% two errors and 22% three errors. The types of errors introduced were insertions, deletions or substitutions in 46, 33 and 21% of the cases, respectively. We repeated this procedure five times, to obtain five distinct versions of the mutated dataset.

Two different partitions of the same dataset (e.g. from two different algorithms), or the partitions of two related datasets (e.g. original versus mutated with the same algorithm), were compared by computing the adjusted Rand index (ARI; Hubert and Arabie, 1985) with the R package ‘clues’.

### 2.5 Accuracy benchmark

For every algorithm, the 1 152 121 ITS1 were clustered together with the 127 577 reference ITS1 previously extracted from the EMBL. The taxa of EMBL entries at the species, genus and family ranks were assigned whenever possible using the NCBI taxonomy. Reference sequences corresponding to environmental samples and uncultured organisms were clustered, but not used to assess the accuracy.

## 3 RESULTS

### 3.1 The DBC454 algorithm

#### 3.1.1 The choice of a metric

The DBC454 algorithm requires that every sequence be represented as a point in an *n*-dimensional space with integer coordinates. Then, the algorithm relies on the computation of the Euclidian distance between two points **X** and **Y**, defined by

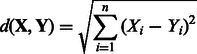



Although sequences are often believed to ‘live in some pseudo-metric space’ (Cai and Sun, 2011), there is no generally accepted manner to encode a sequence as a point in an *n*-dimensional space. Regarding our specific problem with fungal ITS1, which are nucleotide sequences with a length between ∼50 and 300 nt, a relatively simple encoding was investigated and proved a posteriori to be successful.

The simplest encoding is certainly to count the numbers of A, C, G and T of a sequence and to represent it as a point in a four-dimensional space. Obviously, this is a poor encoding, as it is likely that two distinct sequences of the same length can share the same nucleotide composition. However, this principle of encoding can be improved by increasing the number of dimensions, for example, by counting the number of adjacent dinucleotides, which encode a sequence as a point in a 16-dimensional space. The encoding used by DBC454 goes one step further, also counting all the dinucleotide separated by one or two residues. Thus, every sequence is encoded as a point in a 48-dimensional space. For example, the dinucleotide counts for the sequence ATAATA are AT = 2, TA = 2, AA = 1; the counts for dinucleotide separated by one nucleotide are A?A = 2, T?A = 1, A?T = 1; counts for those separated by two nucleotides are A??A = 2, T??T = 1; and all the other counts are zero. In theory, this encoding strategy does not exclude that two unrelated sequences of the same length can share exactly the same encoding. However, we have never observed such a case while working with real data. In contrast, the DBC454 encoding has also some interesting properties, particularly regarding the comparison of pyro-sequences, which are especially prone to insertion/deletion errors that affect repeated stretches of a given nucleotide. The examples given in Table 1 illustrate that the metric featured by DBC454 is, for example, less sensitive to this type of errors than a metric based on pairwise sequence identity. This is also somehow reminiscent of the flowgram comparison of pyro-sequences that have been used by previous authors (Quince *et al.*, 2009).

**Table 1.** Example of distances obtained with the DBC454 metric (see Section 3.1.1) compared with the percent of divergence [100 − percent identity (%ID)], when aligning the wild-type sequence with a sequence in which the GGC (underlined) was replaced with various short sequences

Substitution for GGC	DBC454 distance	100 %ID
AGC	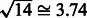	0.820
TGC	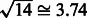	0.820
CGC	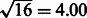	0.820
GGCA	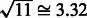	0.810
GGCT	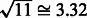	0.810
GGCC	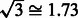	0.810
GGGC	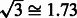	0.810
ATCG	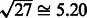	2.440
ATCGATCG	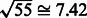	5.510
ATCGATCGATCG	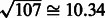	8.400

wild-type: AACGAATGGGTCTTCGGGCCCTTCCAACCCTCAAAACCTGTGGAAGCAAAAGATGTGTTTCGGCGCCGCCGCGCGCCGCATTTATGCAGCGTTATGCTTGTTGTCTGGATTGCAAAGAAATT.

#### 3.1.2 Density-based hierarchical clustering

The DBC454 algorithm depends on two essential parameters: *N*, the minimal number of points that are required to find a valid cluster; and *d*, the maximal distance between two points for them to be connected together. For a given distance threshold *d*, all the points that can be connected together will form a valid cluster if their count reaches, or exceeds, *N*. Taken together, the *N* and *d* parameters implicitly specify the *density constraint* that is placed on the definition of a valid cluster. However, no constraint is placed on the ‘shape’ of a cluster, in that it can be compact and spherical, doughnut shaped or thread shaped. For DBC454, there is no such thing as a cluster ‘center’ as defined for classical hierarchical clustering algorithms, nor of a cluster seed, as implemented in cd-hit-454 and otupipe. Furthermore, as all distances are evaluated, independent runs of the algorithm on the same dataset provide the exact same partition, independently of the sequence order.

**Fig. 2.**
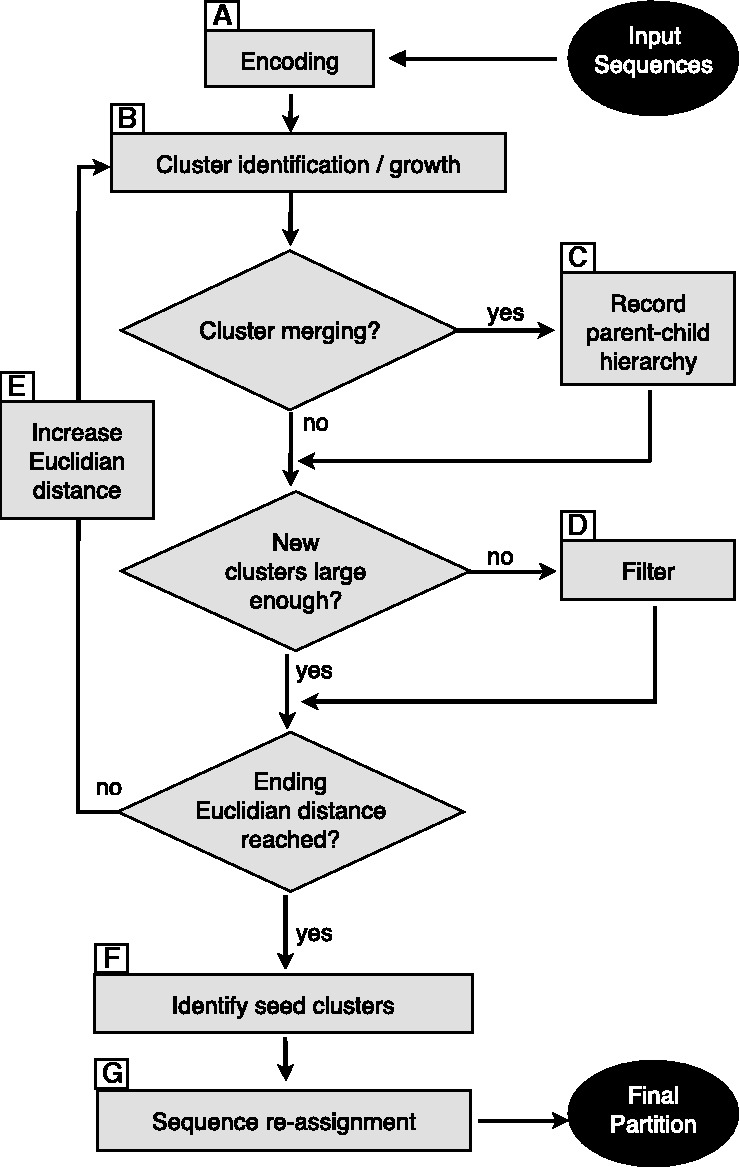
Algorithm flowchart. A: Encoding of the FASTA sequences using dinucleotide counts (see Section 3.1.1 for details). B: Identify new clusters and add sequences to previously identified clusters by single linkage using Euclidian distance cutoff *d*. C: When any previously identified clusters are merging at the distance cutoff *d*, record the cluster content before merging, and update the cluster parent–child hierarchy. D: Remove newly identified clusters containing less than *N* sequences. E: Increase the Euclidian distance cutoff *d* for the next pass. F: Traverse the cluster parent–child merging tree to re-create each cluster discovered in its last state before its merging with another cluster (identification of seed clusters). G: Re-assign sequences to closest seed clusters (see text for detail)

**Fig. 3.**
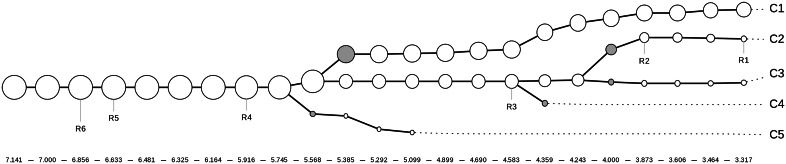
Example of the clustering hierarchy obtained for 5 of the 1199 clusters discovered in the ITS1 dataset. C1–C5: clusters; R1–R6: EMBL reference sequences. For each of the 23 levels, the actual clustering distance used during the computation is indicated at the bottom of the figure. The area of each circle is proportional to the cluster size, ranging from 102 to 2549 sequences. In this example, three clusters are discovered at the lowest level, one at distance 4.359 and one at distance 5.099. The so-called seed clusters are represented in gray. They are the larger clusters found in a branch, just before the first merge event, and play a pivotal role for the post-assignment of the sequences that enter the classification at a higher level

For *d* = 0, no clusters are returned, unless a unique sequence is present at least *N* times in the input dataset. For *d* = ∞, there exists only a single cluster containing all the sequences. A flowchart of the algorithm is presented in Figure 2. The hierarchical clustering starts by providing DBC454 with a low value for *d*, which is progressively increased by a user-specified amount up to the greatest distance value desired. As *d* increases, three observations can be made: (i) new clusters with at least *N* sequences can be discovered; (ii) existing clusters can grow; and (iii) existing clusters can merge. Eventually, a tree of merge events, i.e. a hierarchical clustering, is obtained, and it is possible to retrace the order (according to *d*), in which the clusters have been discovered. Figure 3 presents a typical example of a clustering hierarchy observed in the ITS1 dataset. In addition, it is possible to retrieve the level *d* at which a sequence first entered the classification. This is a convenient way to distinguish the sequences that are representative of the core of a cluster (i.e. they entered the hierarchy at a low *d* value), from outlier sequences that entered at a greater *d* value. When the clustering is stopped at the largest *d* value, the sequences that have not been attributed to any cluster are considered to be noise.

#### 3.1.3 Re-assignment to the final partition

The final number of clusters is taken as equal to the number of leaves found in the hierarchical clustering, independently of the level *d* at which the leaf has appeared. The sequences placed in the leaf clusters account for only a fraction of all the sequences, but represent the core of the classification. The cluster sizes tend to increase while climbing every branch of the tree. The particular clusters found just before their first merging event will be referred to as the *seed clusters* and are shown in gray in Figure 3. At the end of the clustering process, the seed clusters are used to attract and re-assign the sequences that entered the clustering hierarchy in a branch above the first merge event. More precisely, this re-assignment is based on the shortest distance between the sequence to re-assign and every sequence already placed in seed clusters.

When all the sequences have been assigned to a cluster, or left in the noise, DBC454 can optionally process a set of reference sequences supplied by the user, and assigns them to the closest cluster at the proper *d* value. Only reference sequences sufficiently close to an already established cluster are retained, and all the other reference sequences are rejected as noise. Again, the level at which a reference sequence enters the hierarchy is indicative of how close this sequence is to the core of the cluster. When taxonomically curated sequences are used for this optional last step, this allows the annotation of the clusters. By separating the reference sequences (if any) from the main dataset, one ensures that they will not contribute to the cluster discovery and thus alter the results. However, to ease the comparison of the algorithms in the accuracy analysis (see below), this feature was not used (e.g. the whole dataset, including the reference sequences, was clustered all at once).

### 3.2 Number of clusters and percentage of classified sequences

The goal of this study was to identify how the environmental factors influence the fungal diversity at a large geographic scale from a particularly large number of samples. Thus, within this project, it was essential to consider the dominant species and not the rare ones. In other words, clusters should contain a representative number of members to be worth considering in subsequent ecological analyses. In our example of ITS1 sequences generated from 198 geographically different sites, we decided that this minimum number will be 100 (roughly the half of the number of the geographic plots considered in the project). Note that this number corresponds to <0.01% of the total number of sequences. Thus, for DBC454, the minimal number of sequences to seed a cluster was set to 100 (*N* = 100). We have applied the same condition to filter the results of all the other algorithms at the post-processing stage, i.e. all the sequences from clusters smaller than 100 were pooled as noise.

**Fig. 4.**
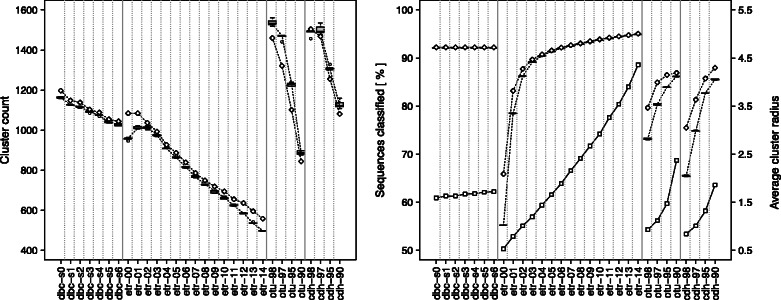
Number of clusters, percentage of sequences classified and average cluster radius for the various algorithms/parameters tested. Left: number of clusters containing at least 100 sequences. Right: percentage of the 1 152 122 input sequences classified in clusters containing at least 100 sequences. The value for the original ITS1 dataset is shown with a white diamond; boxplots show the values obtained for the five mutated datasets. The average cluster radius for the original ITS1 dataset is shown with white squares on the right plot

Figure 4 summarizes the number of clusters that was found by each algorithm, as well as the percentage of classified sequences, i.e. not assigned to the noise. DBC454 and ESPRIT-Tree tend to classify more sequences in a smaller number of clusters than cd-hit-454 and Otupipe. For each algorithm, the number of clusters decreases and the number of classified sequences increases when the clustering parameters are relaxed. This effect is the least pronounced with DBC454. An analysis of the average radius of the clusters discovered shows that DBC454 radii are in the range of those obtained with cd-hit-454 and otupipe. ESPRIT-Tree spans a much larger range of cluster radii.

### 3.3 Robustness analysis

In our view, the most important criterion to evaluate a method of clustering metagenomic data is *robustness*. Ideally, if an experiment is repeated, e.g. if the same samples are re-sequenced, one hopes to retrieve exactly the same cluster structure/OTU definition. In other words, the final partition should be resistant to the errors that actually occur during the sequencing. To measure this quantitatively, we have designed the following numerical benchmark. Five ‘mutated’ datasets were derived from the original dataset, by introducing typical 454 sequencing errors (Huse *et al.*, 2007). See Section 2 for details. We performed the clustering with each one of the five mutated datasets, and compared the partition obtained with the original one. The measure of robustness was given by the ARI, which equals 1 when two partitions are identical and approaches 0 when the partitions are random with respect to each other.

**Fig. 5.**
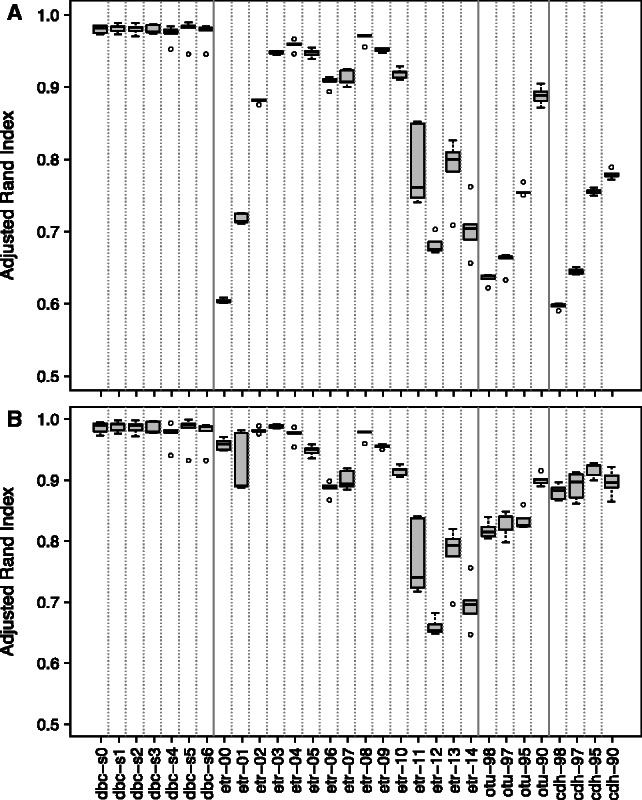
Robustness analysis. Each box represents the ARIs of five comparisons of the original partition with the partitions obtained with the five artificially mutated datasets, for the various algorithms/parameters, as given in Section 2. Above: all clusters with less than 100 members were attributed to noise, and this was treated as a single additional cluster. Below: sequences appearing in the noise either in the original dataset or in the mutated dataset were not taken into account

As explained earlier, we were only interested in clusters containing at least 100 sequences. Thus, before proceeding with the ARI computation, two distinct treatments can be applied to the sequences left in the noise: (i) consider the noise as a single additional cluster; and (ii) reject from the analysis all the sequences that appear in the noise in either one of the two compared partitions. Figure 5A presents the numerical results obtained while considering the noise as a single additional cluster: DBC454 is clearly the most robust method, but ESPRIT-Tree also achieves a good robustness for some intermediate tree-depth values. Figure 5B presents the results, while ignoring the sequences that are unclassified (e.g. appear in the noise) in either one of the two compared partitions. The ARI values are, on average, improved, but the global picture of the comparison of different algorithms remains the same. It should be noted that cd-hit-454 and otupipe are especially sensitive to sequencing errors when their default parameters are used.

### 3.4 Comparison of the different partitions

**Fig. 6.**
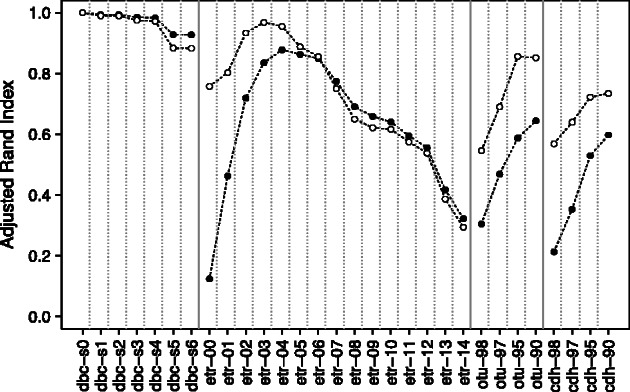
Comparison of the dbc-s0 partition of the ITS1 dataset with the partitions obtained with various algorithms/parameters as described in Section 2. Black plain circle: considering all sequences. White open circle: ignoring sequences that appear in the noise in either of the two partitions compared

Figure 6 shows the results of the comparison of the dbc-s0 partition with the partitions obtained with all the other methods. The DBC454 partitions appear insensitive to the starting distance parameter. Regarding ESPRIT-Tree, the partition etr-04 appears remarkably similar to dbc-s0 (ARI = 0.88 and 0.95 when noise is or is not considered, respectively). Noticeably, etr-04 was previously shown to be one of the most robust partitions produced by ESPRIT-Tree (Fig. 5).

We have conducted a detailed investigation into the discrepancies that exist between the dbc-s0 and etr-04 partitions. Multiple sequence alignments of homologous ITS1 were produced and analyzed using phylogenetic trees and multidimensional scaling (Borg and Groenen, 2005). These strongly suggest to us that both algorithms are essentially producing coherent classifications of the sequences, but sometimes they produce clusters with distinct levels of granularity.

### 3.5 Accuracy analysis

**Fig. 7.**
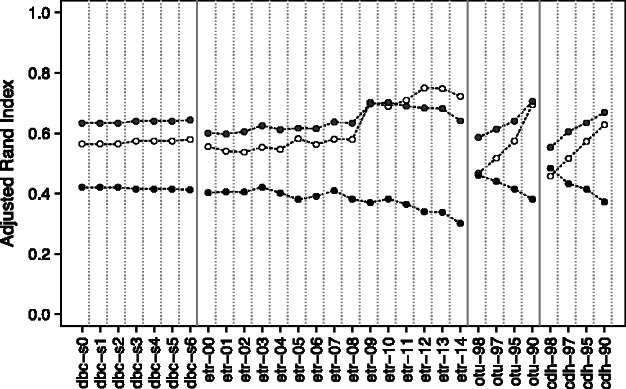
Analysis of the clustering accuracy at the family (white), genus (gray) and species (black) ranks for the various algorithms tested, using the NCBI taxonomy as an external reference

Another round of clustering was performed on an extended dataset consisting of the previously investigated ITS1 sequences plus 127 577 reference ITS1 from the EMBL, for which the taxa at the species, genus and family ranks were obtained from the NCBI taxonomy. It must be noted that most of the reference ITS1 ended up in the noise, i.e. in clusters with less than 100 members, because they were not present in the investigated environmental samples. To evaluate the relative clustering accuracy, we focused on the subset of reference sequences that were always classified by every single method/parameters. This represented a subset of 3365 (62), 3709 (113) and 4022 (1122) sequences (taxa) for family, genus and species, respectively. We used the NCBI taxonomy as the reference partition, and computed the ARI for each partition. Our results (Fig. 7) show that overall, each method behaves similarly, albeit poorly. In particular, the accuracy achieved at the species rank is clearly worse than at the other taxonomic ranks. This might reflect the fact that the ITS1 itself, considering the sequencing errors, is not informative enough to reliably assign the species rank. cdh-98, otu-90 and etr-12 have the best accuracy at the species, genus and family ranks, respectively. However these partitions are not among the most robust, as shown previously. We are aware that such an accuracy analysis is delicate, as it depends both on the investigated dataset and on the quality of the reference taxonomy. This analysis is certainly not devoid of a circularity problem, as part of the reference fungal taxonomy used here was likely established using numerical methods that convey implicit or explicit assumptions on the definition of a fungal taxon.

### 3.6 Computation times

The computational complexity of DBC454 is quadratic with the number of sequences and proportional to the number of levels, in the worst case. The algorithm is fully deterministic and has been parallelized with MPI to run up to 256 CPUs. Actual performance depends on the nature of the dataset. For the dataset reported in this article, all computation times were determined on Intel E5540 Xeon CPU at 2.53 GHz. The computation of the partition of the 1 152 121 input sequences by cd-hit-454 and otupipe both took ∼5 min. The DBC454 computation was executed in parallel on 16 CPUs and took ∼13 min (wall clock time), which actually corresponds to a total of 3.6 h of CPU to generate a hierarchical classification with 23 different levels (i.e. ∼10 min per level). ESPRIT-Tree required ∼9 h of CPU to generate a hierarchical classification with 15 levels.

When considering the mutated dataset, the average computation times increased by 69% (cd-hit-454), 48% (otupipe), 61% (ESPRIT-tree) and 90% (DBC454). This can be explained by the number of unique sequences that are increased in the mutated datasets compared with the original one.

## 4 DISCUSSION

The use of generalized profiles for defining the hyper-variable ITS1 by its highly conserved flanking regions alleviates most of the sensitivity problems encountered with pattern-based search methods (Bellemain *et al.*, 2010), including sequencing errors, such as indels. In addition, this permits the recovery of all the ITS1 from the EMBL, irrespective of the particular primers that have been used by the original authors.

DBC454 relies on an arbitrarily defined encoding of every sequence as a point in a 48-dimensional space. When this encoding is used for hierarchical density-based clustering, an especially robust partition of the input sequences is obtained, which a posteriori corroborates its relevance.

DBC454 uses density-based clustering to identify groups of related sequences that are naturally dense in the input dataset. As a density-based method, DBC454 places no constraints on the cluster shape and radius, and has no concept of a cluster center. In contrast to all the other algorithms considered here, which remove the redundancy among the sequences as an initial step, DBC454 explicitly uses this information for the clustering (see the note on ESPRIT-Tree below). One of the unintuitive aspects of density-based clustering is that its performance tends to improve with the size of the input dataset, contrary to many other clustering approaches. Our results show that this approach leads to especially robust partitions without being detrimental to the overall accuracy.

This study demonstrates that DBC454 and ESPRIT-tree (at a carefully chosen level) are currently the two most robust methods for the definition of OTUs in the dataset we have studied. The partitions produced by these two methods are only slightly different and essentially compatible. The key observation here is that DBC454 and ESPRIT-Tree rely on completely different algorithmic approaches. This indicates that both methods are actually capable of ‘recognizing’ the same structure in the input dataset, which is an encouraging result regarding the future reproducibility of the experiments in the field of metagenomics. It must be noted that the ESPRIT-Tree algorithm also includes a ‘density-sensitive’ computation step, i.e. the weighting scheme that is used to update the probabilistic sequences at level greater than 0 (Cai and Sun, 2011). This might help to explain the convergent results obtained for DBC454 and ESPRIT-Tree.

Recently, much attention has been placed on the benefits of ‘denoising’, i.e. correct the suspected sequencing errors of the pyro-sequences before performing the taxonomy-independent analysis itself (Bonder *et al.*, 2012). Considering their particular sensitivity to the sequencing errors demonstrated here, this recommendation obviously makes sense when cd-hit-454, otupipe or ESPRIT-Tree (at the lowest level) is used for the clustering analysis. However, the introduction of an additional pre-processing step implies the optimization of additional parameters and has a cost in terms of CPU, which is currently prohibitive for large datasets. Instead of requiring two successive steps (‘denoising’ followed by clustering), we have shown here that the clustering process itself can be used at the same time to produce a robust partition (insensitive to sequencing errors) and to identify the outlier sequences, i.e. those that were pooled as ‘noise’ in this article.
